# Supplementary material for: Quantifying the mental health and economic impacts of prospective Universal Basic Income schemes among young people in the UK: a microsimulation modelling study
Source: BMJ Open. 2023 Oct 4;13(10):e075831. doi: 10.1136/bmjopen-2023-075831 (PMC10551934; doi:10.1136/bmjopen-2023-075831)
Supplement: Author's manuscript [file bmjopen-2023-075831.draft_revisions.pdf]

BMJ Open is committed to open peer review. As part of this commitment we make the peer review history of every article we publish publicly available.

When an article is published we post the peer reviewers' comments and the authors' responses online. We also post the versions of the paper that were used during peer review. These are the versions that the peer review comments apply to.

The versions of the paper that follow are the versions that were submitted during the peer review process. They are not the versions of record or the final published versions. They should not be cited or distributed as the published version of this manuscript.

BMJ Open is an open access journal and the full, final, typeset and author-corrected version of record of the manuscript is available on our site with no access controls, subscription charges or pay-per-view fees (http://bmjopen.bmj.com).

If you have any questions on BMJ Open's open peer review process please email info.bmjopen@bmj.com

Quantifying the mental health and economic impacts of prospective Universal Basic Income schemes among young people in the UK: a microsimulation modelling study

Journal:	*BMJ Open*
Manuscript ID	bmjopen-2023-075831
Article Type:	Original research
Date Submitted by the Author:	19-May-2023

Complete List of Authors:	Chen, Tao; University of York<br>Reed, Howard; Landman Economics Ltd; Northumbria University, Department of Social Sciences<br>Mujica, Fiorella Parra; Erasmus University Rotterdam<br>Johnson, Elliott; Northumbria University, Department of Social Sciences<br>Johnson, Matthew; Northumbria University, Department of Social Sciences<br>O'Flaherty, M; University of Liverpool, Department of Public Health, Policy & Systems<br>Collins, Brendan; University of Liverpool, Department of Public Health, Policy and Systems<br>Kypridemos, Christodoulos ; University of Liverpool, Department of Public Health, Policy & Systems

Keywords:	MENTAL HEALTH, Depression & mood disorders < PSYCHIATRY, Anxiety disorders < PSYCHIATRY, PUBLIC HEALTH

SCHOLARONE™
Manuscripts

I, the Submitting Author has the right to grant and does grant on behalf of all authors of the Work (as defined in the below author licence), an exclusive licence and/or a non-exclusive licence for contributions from authors who are: i) UK Crown employees; ii) where BMJ has agreed a CC-BY licence shall apply, and/or iii) in accordance with the terms applicable for US Federal Government officers or employees acting as part of their official duties; on a worldwide, perpetual, irrevocable, royalty-free basis to BMJ Publishing Group Ltd ("BMJ") its licensees and where the relevant Journal is co-owned by BMJ to the co-owners of the Journal, to publish the Work in this journal and any other BMJ products and to exploit all rights, as set out in our licence.

The Submitting Author accepts and understands that any supply made under these terms is made by BMJ to the Submitting Author unless you are acting as an employee on behalf of your employer or a postgraduate student of an affiliated institution which is paying any applicable article publishing charge ("APC") for Open Access articles. Where the Submitting Author wishes to make the Work available on an Open Access basis (and intends to pay the relevant APC), the terms of reuse of such Open Access shall be governed by a Creative Commons licence – details of these licences and which Creative Commons licence will apply to this Work are set out in our licence referred to above.

Other than as permitted in any relevant BMJ Author's Self Archiving Policies, I confirm this Work has not been accepted for publication elsewhere, is not being considered for publication elsewhere and does not duplicate material already published. I confirm all authors consent to publication of this Work and authorise the granting of this licence.

For peer review only

Quantifying the mental health and economic impacts of prospective Universal Basic Income schemes among young people in the UK: a microsimulation modelling study

Authors:

Tao Chen[1], tao.chen@york.ac.uk

Howard Reed[2,3], howard.r.reed@gmail.com

Fiorella Parra Mujica[4], fiorellaparra@gmail.com

Elliott Johnson[3], elliott.johnson@northumbria.ac.uk

Matthew Johnson[3], matthew7.johnson@northumbria.ac.uk

Martin O'Flaherty[5], moflaher@liverpool.ac.uk

Brendan Collins[5], brenc@liverpool.ac.uk

Chris Kypridemos[5], C.Kypridemos@liverpool.ac.uk

1. Centre for Health Economics, University of York
2. Landman Economics Ltd, http://www.landman-economics.co.uk/
3. Department of Social Sciences, Northumbria University, UK
4. Erasmus School of Health Policy and Management (ESHPM), Erasmus University Rotterdam, Netherlands
5. Department of Public Health, Policy & Systems, University of Liverpool, UK

For peer review only

Word Count: 3,062

Corresponding author: Chris Kypridemos, PhD, Department of Public Health, Policy & Systems, Institute of Population Health, Whelan Building, Quadrangle, LIVERPOOL, L69 3GB, UK

Funding

This research was funded by a Wellcome Trust Discretionary Award (223553/Z/21/Z): 'Assessing the prospective impacts of Universal Basic Income on anxiety and depression among 14–24-year-olds'. The funder had no role in study design, data collection and analysis, decision to publish, or preparation of the manuscript.

Conflict of interests

BC works part-time for Welsh Government; this does not represent any views of Welsh Government. All other co-authors declare no conflict of interests.

For peer review only

Abstract:

Background and objective

We aimed to quantify the long-term impact of Universal Basic Income(UBI) on mental health among young people in England.

Design

We produced a discrete-time dynamic stochastic microsimulation that models a close-to-reality open cohort of synthetic individuals between 2010 and 2030 based on data from the Office for National Statistics and the Understanding Society UK Household Longitudinal Study. Three UBI scheme scenarios were simulated: Scheme 1 – Starter (per week): £41 per child; £63 per adult over 18 and under 65; £190 per adult aged 65+; Scheme 2 – Intermediate (per week): £63 per child; £145 per adult under 65; £190 per adult aged 65+; Scheme 3 – Minimum Income Standard level (per week): £95 per child; £230 per adult under 65; £230 per adult aged 65+.

Setting and Participants: Synthetic population for England.

Outcome Measures: Cases of anxiety and depression prevented or postponed and cost savings compared with the existing system.

Results

Scheme 1 could prevent or postpone 200,000 (95% uncertainty interval: 180,000 - 210,000) cases of anxiety and depression between 2010 and 2030). This would increase to 420,000 (400,000 - 440,000) for Scheme 2 and 550,000 (520,000 - 570,000) for Scheme 3. Assuming that 50% of cases are diagnosed and treated, Scheme 1 could save £330m (£280m - £290m) health costs, over the same period, with Scheme 2 (£710 million [£640m - £790m]) or Scheme 3 (£930 million [£850m - £1000m]) producing more considerable savings. Overall, the total cost savings (including NHS, personal social services and patients' related costs) would range from £1.5 billion (£1.2b - £1.8b) for Scheme 1 to £4.2 billion (£3.7b - £4.6b) for Scheme 3.

Conclusion

Our modelling suggests that UBI could substantially benefit young people's mental health and would produce substantial health cost savings.

For peer review only

Keywords: Universal Basic Income; Mental Health; Prevention; Microsimulation Modelling

Strengths and limitations of this study

- First study to model impacts of universal basic income on mental health in young people
- Results suggest universal basic income can have a substantial impact on incidence of anxiety and depression and related health costs, which is useful for policy makers
- Limited to pre-pandemic data so impacts of cost of living crisis and other changes since the pandemic may change the results
- Does not include impact of people moving into higher tax brackets ('fiscal drag')

For peer review only

Introduction

There is a crisis in mental health among young people, which will have long-term impacts on well-being and development. Between 1995 and 2014, the proportion of 16- to 24-year-olds in the United Kingdom (UK) reporting a longstanding mental health condition increased from 0.6% to 5.9% [1]. A meta-analysis including 11 high-income countries indicated that one in eight children have mental disorders[2]. Unfortunately, this problem may have been exacerbated since the recent austerity period in the UK[3] and further magnified during the COVID pandemic[4]. Currently, it is estimated that childhood mental disorders are the leading cause of childhood disability globally[5] and incur considerable social and economic burdens to the healthcare system and families[6 7].

Previous studies have found that adverse economic conditions could negatively affect mental health in children and young people[8-10]. Our previous analysis of Understanding Society data for young people aged 14-24 from UK households went further, showing a dose-response effect[11]. Young people living in households within the lowest net equivalised income quintile group had a higher probability than the second lowest quintile group of reporting clinically significant symptoms of anxiety and depression; the second lowest had a higher probability than the middle quintile group and so on up the income scale.

To address this public health concern, many approaches have been proposed to promote mental health and prevent mental disorders[12 13]. However, these reactive policies have often focused on individual-level interventions such as improving coping strategies and increasing the efficiency of services. At the same time, interest is growing in addressing the social causes of anxiety and depression. A large body of evidence indicates that social determinants strongly affect those conditions: income, wealth, education, social capital and opportunity[14-17]. One proposed means of addressing these issues, which is increasingly gaining support from various organisations, policymakers and politicians, is Universal Basic Income (UBI), a largely

For peer review only

unconditional, regular payment to all permanent residents to support basic needs. Johnson and colleagues have set out a theoretical model of its impact that indicates that UBI can mitigate social determinants of health by reducing poverty, mitigating inequality and fostering long-term, health-promoting behaviour[18].

Previous modelling has examined the potential costs and benefits of mental health interventions to prevent or treat anxiety, depression, bipolar disorder, and suicide among adolescents[13] or by comparing cognitive behavioural therapy and selective serotonin reuptake inhibitors for major depression in children and adolescents[19]. However, no study assessed the long-term impact of UBI on mental health in children and young people.

This study aimed to quantify the potential impacts of three prospective UBI schemes on the mental health of young people and the associated economic burden during the 2010-2030 period in the UK.

Methods

Our study used two microsimulations in a hybrid serial modelling arrangement to simulate three UBI scheme scenarios and estimate the prevalence of anxiety and depression and consequent deaths under the counterfactual net equivalised household income distributions.

The three UBI scheme scenarios were broadly designed to provide pathways towards attaining the Minimum Income Standard (MIS) with income distributions microsimulated using the Landman Economics Tax-Transfer Model (first microsimulation in the serial arrangement). MIS is the income needed by different types of households to reach a socially acceptable living standard, as determined by members of the public with support from experts[20]. The three schemes are detailed below:

Scheme 1 – Starter (per week): £41 per child; £63 per adult over 18 and under 65; £190 per adult aged 65+

For peer review only

Scheme 1 is fiscally neutral in static terms and does not include savings and returns from investment elsewhere as a result of its introduction. It is affordable under any definition. No additional funding from the Exchequer and no net increase in taxation would be required.

Scheme 2 – Intermediate (per week): £63 per child; £145 per adult under 65; £190 per adult aged 65+

Scheme 2 is a mid-point between the lower and higher levels. It is not fiscally neutral but could be funded by a range of means.

Scheme 3 – MIS level (per week): £95 per child; £230 per adult under 65; £230 per adult aged 65+

Scheme 3 ensures that all families reach the MIS level. It has a substantial up-front cost but can be funded by a range of means.

Each of the above schemes is intended to meet the following conditions:

1. UBI would be paid to eligible residents without condition, raising the incomes of the lower income groups.
2. UBI would reduce the percentage gap between the top and bottom income groups through fiscal reform, be high enough to make a material difference in people's lives and raise the level of universality in the social security system, thus reducing reliance on means-testing.
3. UBI would be affordable (although this depends on how this is defined);
4. UBI would minimise losses for low-income households, minimise the amount of disruption involved in moving to a new income support system, and enjoy broad public support. For instance, these schemes have been found to enjoy support among critical "red wall" voters[21].

For peer review only

Household income modelling

The Landman Economics Tax Transfer Model was used with Waves 1 to 10 (inclusive) of Understanding Society data to microsimulate the UBI payments in Schemes 1, 2 and 3 and corresponding packages of increases to tax (income tax and National Insurance contributions) required to achieve fiscal balance for each scheme, taking into account reductions in payments of existing benefits[22]. The main non-means-tested benefits in the UK benefits system (Child Benefit and the State Pension) are replaced by the UBI in each scheme. UBI payments are counted as unearned income to calculate Universal Credit (a means-tested transfer payment for people on low incomes in the UK benefits system). So UBI payments replace Universal Credit payments one-for-one for low-income individuals (although a small disregard is applied for schemes 1 and 2 so that low-income individuals and families are better off under UBI than the baseline system. In Scheme 3, UBI payments are sufficiently high that no disregard is necessary). Note that fiscal balancing for each scheme (i.e. ensuring that the increase in tax revenue approximately matches the cost of UBI expenditure, net of any reductions in other benefits) is done statically in the model, not taking account of any behavioural changes in response to the receipt of UBI payments.

The level of payments in each of the schemes is based on existing analysis by Reed et al.[22] using data from the UK Family Resources Survey (FRS) for the 2019/20 fiscal year. This overlaps with the interview dates for Understanding Society Wave 10, so the same level of payments is used for Wave 10 as for the FRS analysis. For earlier waves of Understanding Society, the UBI payments are deflated using the UK Consumer Prices Index. The income tax and National Insurance increases are adjusted in each wave to ensure approximate fiscal balance.

Health and disease costs impact modelling

The second microsimulation in the serial arrangement was a discrete-time dynamic stochastic microsimulation that used the output of the first microsimulation (changes in the distribution

For peer review only

of the equivalised household income) and translated into changes in the prevalence of anxiety and depression and the consecutive disease costs. Specifically, the second microsimulation models a close-to-reality open cohort of synthetic individuals (starting at 90,000) representing individuals aged 14-24 in the UK between 2010 and 2030. Their rates of fertility, mortality, and migration were driven by Office for National Statistics estimates and projections[23]. At the same time, ethnicity, whether born in the UK, highest educational attainment and marital status were informed by waves 1-10 of the Understanding Society: the UK Household Longitudinal Study[24]. We simulated the prevalence of anxiety and depression using the SF-12 Mental Component Summary measure with a clinical threshold score of ≤45.6. Our simulation was based on all attributes above, including equivalised household income, based on evidence from Parra-Mujica et al.[11], assuming a causal relationship between income and anxiety and depression and total risk reversibility. For all the attributes described above, we fitted logistic regression models to the Understanding Society data and predicted from them to allocate the attributes of the synthetic individuals. We further assumed that the observed increasing trend of anxiety and depression prevalence would plateau after 2019 to avoid unrealistic increase over time. We did similar for the equivalised household income trends post 2019.

In our microsimulation, we also modelled reductions in cause-excess deaths based on relative risks (RR) identified in observational data from Denmark by Meier et al. [25] To account for the fact that our case definition of anxiety and depression, based on a self-reported measure (SF-12 MCS), might include less severe cases compared to the clinically diagnosed cases in the study by Meier at al. and reflect the uncertainty of the issue we formed a beta-PERT distribution[26] based on estimates from the study mentioned above. We used the low 95% confidence interval of the fully adjusted all-cause mortality RR (1.56) reported for individuals diagnosed with anxiety disorders as the minimum for our beta-PERT distribution, the anxiety disorders RR for ages <30 (4.4) as the mode of the distribution, and the dual diagnosis of

For peer review only

anxiety and depression RR for ages < 30 (6.9) as the maximum of the distribution. These estimates were comparable to published estimates from Sweden, albeit using slightly different case definitions[27].

Costs

Costs for anxiety and depression treatment were informed by the usual care arm of the CADET randomised control trial[28]. We estimated and report two different cost perspectives 1) the UK NHS and Personal Social Services (Third Party Payer) perspective, and 2) A broader perspective that included resource use from primary/Community Care (e.g., GP, Mental Health worker, Social worker), Secondary Care (e.g., Hospital admissions, Psychiatric rehab ward, Outpatient appointment, social care (e.g., Daycare centre, drop in a club), informal care from friends/relatives (e.g., Hours per week help from friends/relatives), patient other costs (e.g., OTC medications, Travel costs) to estimate the total cost of anxiety and depression. We inflated all costs to mid-2015 British pounds using the Consumer Price Index and did not apply an annual discount rate for costs occurring in the past or future. Finally, we used the reported mean and standard deviation of the costs to form Gamma distributions using the method of moments and capture the uncertainty of the inputs. We further assumed that only half of the synthetic individuals that reported symptoms of anxiety or depression would seek treatment and thus incur healthcare costs. This assumption was roughly informed by the Adult Psychiatric Morbidity Survey[29].

Uncertainty and sensitivity analysis

In all reported figures, we ensured that we captured the uncertainty of the outputs. The microsimulation used a second-order Monte Carlo with 200 iterations for the outer loop and ~90,000 iterations for the inner loop to propagate the uncertainty of the inputs to the outputs[30]. We summarised the uncertainty of the outputs by reporting the median and 95% Uncertainty Interval (UI) of their respective distributions. The three main sources of uncertainty in the model are 1) the strength of the relation between equivalised household income and anxiety

For peer review only

and depression, 2) the excess mortality risk from anxiety and depression, 3) the disease costs, and 4) the individual heterogeneity from the modelled attributes of the synthetic individuals.

The outputs from the model are case-years of anxiety and depression prevented or postponed (CYPP), deaths prevented or postponed (DPP), disease costs from the NHS and personal social services perspective, and total disease costs.

Results

The UK population between 14 and 24 years old was projected to increase from approximately 9 million in 2010 to 9.6 million in 2030. Of those, about 2 million would experience symptoms of anxiety and depression in 2010; this was projected to more than double to 4.1 million by 2030. Anxiety and depression were more prevalent in females, about 46%, versus about 30% in males, and their prevalence increased with age. It was also slightly more prevalent among non-White and those born in the UK.

The model estimated that approximately 200,000 (95% UI: 180,000 - 210,000) cases of anxiety and depression could be prevented or postponed in Scheme 1 from 2010-2030. The effectiveness would increase to 420,000 (95% UI: 400,000 - 440,000) for Scheme 2 and 550,000 (95% UI: 520,000 - 570,000) for Scheme 3. In relative terms, these represent approximately 0.028% (95% UI: 0.026% - 0.030%) of all case years with anxiety and depression for Scheme 1, 0.059% (95% UI: 0.056% - 0.063%) for Scheme 2 and 0.077% (95% UI: 0.074% - 0.081%) for Scheme 3. Correspondingly, 110 (95% UI: 0 - 430), 320 (95% UI: 0 - 640) and 420 (95% UI: 100 - 770) deaths would be prevented or postponed for the three Schemes, respectively.

For peer review only

Table 1 shows the NHS and personal social services cost savings and total cost savings. Overall, the total cost saving, including NHS, personal social services and patient-related costs, would range from £1.5 billion (£1.2b - £1.8b) for Scheme 1 to £4.2 billion (£3.7b - £4.6b) for Scheme 3.

Table 1: Modelling results estimating disease cost savings from different perspectives

Schemes	NHS and personal social services cost savings over 2010-2030, assuming 50% of cases diagnosed and treated	Total cost savings over 2010-2030, assuming 50% of cases diagnosed and treated
**Scheme 1**	£330 million (£280m - £290m)	£1.5 billion (£1.2b - £1.8b)
**Scheme 2**	£710 million (£640m - £790m)	£3.2 billion (£2.8b - £3.6b)
**Scheme 3**	£930 million (£850m - £1000m)	£4.2 billion (£3.7b - £4.6b)

Discussion

Our findings indicate the mental health impact that UBI could have on a specific age group through a pathway of increased income. Despite the limited scope of the present modelling study, it is clear that the potential is substantial and significant. Over 21 years, 200 to 550 thousand cases of anxiety and depression could be prevented or postponed, saving £330m to £930m in health and social services costs.

Strengths and Limitations

To our knowledge, this is the first study to model the health and disease cost impact of UBI among young people. Previous modelling mainly focused on assessing mental health prevention through trial-based economic evaluation[19 28] but was subject to inadequate patient

For peer review only

follow-up and not capturing the final health outcomes. However, our model-based design is fundamental in an economic evaluation of mental health prevention due to its advantages, including the ability to consider all relevant prospective policy alternatives, to include evidence that is not often collected in trials, and to extrapolate beyond the usually short-term horizon of empirical studies[31].

Our modelling exercise assumes that low income is causally related to anxiety and depression and that increasing income can fully reverse the risk. The association between income and mental health has been shown in experimental and observational studies[10 15]. However, the heterogeneity of cash transfer schemes and other policies intended to redistribute income and the heterogeneity of reported mental health outcomes make evidence synthesis difficult. Large, representative trials of UBI that capture comprehensive and comparable data in the real world are crucial[14].

Our modelling exercise has some limitations. First, when setting the UBI payment levels and the income tax thresholds in the reform schemes, we assumed that both are CPI-uprated between Understanding Society Waves 1 and 10. This means that UBI payments for each adult and child remain constant in real terms from year to year. We made the same assumption about tax. However, this fails to account for the fact that real earnings grew in most years between 2010 and 2019, resulting in a process known as "fiscal drag" (taxpayers tending to move into higher marginal rate brackets) that would gradually decrease the impact of the UBI schemes. To minimise this bias and considering the turbulent period since 2020, we did not model trends in equivalised household income post 2019. Second, all the data we used were from the years before the COVID-19 pandemic and the post-pandemic cost-of-living crisis. Therefore, the trends we modelled may not be indicative of the post-pandemic period up to 2030. Specifically, the pandemic may increase the prevalence of anxiety and depression in the population and further limit access to appropriate treatments and support. Furthermore, the post-pandemic

For peer review only

cost-of-living crisis and the high-inflation period may compress family incomes and accelerate the mental health crisis. These limitations make our modelled estimates conservative and research on UBI policies more relevant than ever.

Future Research

In the future, it is imperative to develop models that comprehensively capture the health impact of income changes across the entire population and all major disease types. These models should incorporate quality of life measures such as quality-adjusted life years (QALYs) gained, and their value could be evaluated based on NICE or UK Treasury valuations. Such an approach would enable assessment of the potential cost savings that could be achieved through improved health outcomes under a UBI policy. Moreover, the additional equity and well-being benefits of UBI, which are not fully captured through a 'burden of disease' perspective, may further offset some of the financial burdens associated with implementing such policies.

Policy Implications

Regarding policy implications, the present study provides evidence that UBI can produce health benefits for young people over a medium-term time horizon. This is useful evidence for the basic income trial currently underway in Wales, in which care leavers are offered a basic income of £1,600 per month (higher than Scheme 3 in the present study)[32]. Care leavers have rates of mental health problems that are up to six times that of the non-care exposed population[33], so there is potential for a basic income for care leavers to have a greater relative effect in this group, depending on how much mental health problems are related to income in this population. There is also a basic income pilot in Santa Clara County (California, USA), where people leaving foster care at age 25 receive $1000 a month. Including common outcome measures such as SF-12 in these real-world pilots would provide further data to compare with the results of this study and enable further microsimulation modelling.

For peer review only

The concept of a guaranteed minimum income the state provides to all permanent residents is gaining traction across the political spectrum. Even the conservative-leaning UK think tank Bright Blue recently called for "the establishment of a new 'minimum living' income"[34], although largely within the UK welfare system as it is currently constituted. A scoping review of the public health effects of interventions resembling basic income found "modest to strong positive effects on several health outcomes, including low birth weight, infant obesity, adult and child mental health, service use, and nutrition"[35]. An evidence synthesis based on several studies of basic income programmes found that, overall, basic income improved mental health, with mediating factors being increased free time, hope for the future, and reduced stigma[36].

Conclusions

In summary, the present study suggests that UBI could substantially improve mental health in young people, reduce costs related to the NHS, personal social services and patients, and reduce premature mortality. These findings add to the growing body of evidence supporting the potential for UBI to improve population health.

Author Statement

MJ and EJ conceptualised the study; TC and FPM analysed the data; HR developed and performed the UBI modelling; CK developed and performed the health modelling and supervised all the analysis; TC produced the first draft; all authors interpreted the results and critically revised the manuscript.

Patient and Public Involvement Statement

There was no patient and public involvement in this study.

Data statement

This is a modelling study that generated no new empirical data. Understanding Society is available through the UK data service (https://ukdataservice.ac.uk/).

For peer review only

References:

1. Pitchforth J, Fahy K, Ford T, et al. Mental health and well-being trends among children and young people in the UK, 1995-2014: analysis of repeated cross-sectional national health surveys. *Psychol Med* 2019;49(8):1275-85. doi: 10.1017/S0033291718001757 [published Online First: 2018/09/12]
2. Barican JL, Yung D, Schwartz C, et al. Prevalence of childhood mental disorders in high-income countries: a systematic review and meta-analysis to inform policymaking. *Evid Based Ment Health* 2022;25(1):36-44. doi: 10.1136/ebmental-2021-300277 [published Online First: 2021/07/21]
3. Thomson RM, Katikireddi SV. Mental health and the jilted generation: Using age-period-cohort analysis to assess differential trends in young people's mental health following the Great Recession and austerity in England. *Soc Sci Med* 2018;214:133-43. doi: 10.1016/j.socscimed.2018.08.034 [published Online First: 20180829]
4. Ma L, Mazidi M, Li K, et al. Prevalence of mental health problems among children and adolescents during the COVID-19 pandemic: A systematic review and meta-analysis. *J Affect Disord* 2021;293:78-89. doi: 10.1016/j.jad.2021.06.021 [published Online First: 2021/06/27]
5. Erskine HE, Moffitt TE, Copeland WE, et al. A heavy burden on young minds: the global burden of mental and substance use disorders in children and youth. *Psychol Med* 2015;45(7):1551-63. doi: 10.1017/S0033291714002888 [published Online First: 2014/12/24]
6. Lim KL, Jacobs P, Ohinmaa A, et al. A new population-based measure of the economic burden of mental illness in Canada. *Chronic Dis Can* 2008;28(3):92-8. [published Online First: 2008/03/18]
7. Fineberg NA, Haddad PM, Carpenter L, et al. The size, burden and cost of disorders of the brain in the UK. *J Psychopharmacol* 2013;27(9):761-70. doi: 10.1177/0269881113495118 [published Online First: 2013/07/26]
8. Golberstein E, Gonzales G, Meara E. How do economic downturns affect the mental health of children? Evidence from the National Health Interview Survey. *Health Econ* 2019;28(8):955-70. doi: 10.1002/hec.3885 [published Online First: 2019/06/06]
9. Pfoertner TK, Rathmann K, Elgar FJ, et al. Adolescents' psychological health complaints and the economic recession in late 2007: a multilevel study in 31 countries. *Eur J Public Health* 2014;24(6):961-7. doi: 10.1093/eurpub/cku056 [published Online First: 2014/05/27]
10. Thomson RM, Igelstrom E, Purba AK, et al. How do income changes impact on mental health and wellbeing for working-age adults? A systematic review and meta-analysis. *Lancet Public Health* 2022;7(6):e515-e28. doi: 10.1016/S2468-2667(22)00058-5 [published Online First: 2022/06/07]
11. Parra-Mujica F, Johnson, E.A., Cookson, R., Reed, H. & Johnson, M.T. Understanding the relationship between socioeconomic status, income and mental health among 16- to 24-year-olds: Analysis of 10 waves (2009-2020) of Understanding Society to enable modelling of income interventions. *working paper* 2022
12. Dunning DL, Griffiths K, Kuyken W, et al. Research Review: The effects of mindfulness-based interventions on cognition and mental health in children and adolescents - a meta-analysis of randomized controlled trials. *J Child Psychol Psychiatry* 2019;60(3):244-58. doi: 10.1111/jcpp.12980 [published Online First: 2018/10/23]
13. Skeen S, Laurenzi CA, Gordon SL, et al. Adolescent Mental Health Program Components and Behavior Risk Reduction: A Meta-analysis. *Pediatrics* 2019;144(2) doi: 10.1542/peds.2018-3488 [published Online First: 2019/07/03]
14. Johnson EA, Johnson, M.T., Kypridemos, C., Villadsen, A. & Pickett, K.E. Designing a generic, adaptive protocol resource for the measurement of health impact in cash transfer trials'. *Research Square* 2022
15. Romero J, Esopo, K., McGuire, J. & Haushofer. The Effect of Economic Transfers on Psychological Well-Being and Mental Health. *working paper* 2021

For peer review only

16. Kromydas T, Thomson RM, Pulford A, et al. Which is most important for mental health: Money, poverty, or paid work? A fixed-effects analysis of the UK Household Longitudinal Study. *SSM-population Health* 2021;15:100909.
17. Thomson RM, Igelström E, Purba AK, et al. How do income changes impact on mental health and wellbeing for working-age adults? A systematic review and meta-analysis. *The Lancet Public Health* 2022;7(6):e515-e28. doi: 10.1016/s2468-2667(22)00058-5
18. Johnson MT, Johnson EA, Nettle D, et al. Designing trials of Universal Basic Income for health impact: identifying interdisciplinary questions to address. *J Public Health (Oxf)* 2022;44(2):408-16. doi: 10.1093/pubmed/fdaa255
19. Haby MM, Tonge B, Littlefield L, et al. Cost-effectiveness of cognitive behavioural therapy and selective serotonin reuptake inhibitors for major depression in children and adolescents. *Aust N Z J Psychiatry* 2004;38(8):579-91. doi: 10.1080/j.1440-1614.2004.01421.x [published Online First: 2004/08/10]
20. Davis A, Hirsch, D., Padley, M. & Shepherd, C. A Minimum Income Standard for the United Kingdom in 2021: York: Joseph Rowntree Foundation 2021.
21. Johnson M, Nettle D, Johnson E, et al. Winning the vote with a universal basic income: Evidence from the 'red wall', 2022.
22. Reed HR, Johnson MT, Lansley S, et al. Universal Basic Income is affordable and feasible: evidence from UK economic microsimulation modelling. *Journal of Poverty and Social Justice* 2023;31(1):146-62. doi: 10.1332/175982721X16702368352393
23. 2020-based interim national population projections - Office for National Statistics [
24. Research IfSaE. Understanding Society, 2022.
25. Meier SM, Mattheisen M, Mors O, et al. Increased mortality among people with anxiety disorders: total population study. *Br J Psychiatry* 2016;209(3):216-21. doi: 10.1192/bjp.bp.115.171975 [published Online First: 2016/07/09]
26. Clark CE. Letter to the editor-the PERT model for the distribution of an activity time. *Operations Research* 1962;10(3):405-06. doi: 10/bg3bp2
27. Leone M, Kuja-Halkola R, Leval A, et al. Association of Youth Depression With Subsequent Somatic Diseases and Premature Death. *JAMA Psychiatry* 2021;78(3):302-10. doi: 10.1001/jamapsychiatry.2020.3786 [published Online First: 2020/12/10]
28. Green C, Richards DA, Hill JJ, et al. Cost-effectiveness of collaborative care for depression in UK primary care: economic evaluation of a randomised controlled trial (CADET). *PLoS One* 2014;9(8):e104225. doi: 10.1371/journal.pone.0104225 [published Online First: 2014/08/15]
29. Digital N. Adult Psychiatric Morbidity Survey: Survey of Mental Health and Wellbeing, England, 2014. 2014 [
30. Koerkamp BG, Stijnen T, Weinstein MC, et al. The combined analysis of uncertainty and patient heterogeneity in medical decision models. *Medical Decision Making* 2011;31(4):650-61. doi: 10/cdf2wf
31. Ha NT, Huong NT, Anh VN, et al. Modelling in economic evaluation of mental health prevention: current status and quality of studies. *BMC Health Serv Res* 2022;22(1):906. doi: 10.1186/s12913-022-08206-9 [published Online First: 2022/07/14]
32. Government W. Basic income pilot for care leavers: overview of the scheme 2023 [updated 2023-02-15.
33. Butterworth S, Singh SP, Birchwood M, et al. Transitioning care-leavers with mental health needs: 'they set you up to fail!'. *Child Adolesc Ment Health* 2017;22(3):138-47. doi: 10.1111/camh.12171 [published Online First: 20160629]
34. Sarygulov A, Arslanagić-Wakefield P. Building up: The future of social security. London, UK: bright blue, 2023.
35. Gibson M, Hearty W, Craig P. The public health effects of interventions similar to basic income: a scoping review. *The Lancet Public Health* 2020;5(3):e165-e76. doi: 10.1016/S2468-2667(20)30005-0

For peer review only

36. Wilson N, McDaid S. The mental health effects of a Universal Basic Income: A synthesis of the evidence from previous pilots. *Soc Sci Med* 2021;287:114374. doi: 10.1016/j.socscimed.2021.114374 [published Online First: 20210907]

For peer review only

Reporting checklist for cohort study.

Based on the STROBE cohort guidelines.

Instructions to authors

Complete this checklist by entering the page numbers from your manuscript where readers will find each of the items listed below.

Your article may not currently address all the items on the checklist. Please modify your text to include the missing information. If you are certain that an item does not apply, please write "n/a" and provide a short explanation.

Upload your completed checklist as an extra file when you submit to a journal.

In your methods section, say that you used the STROBE cohortreporting guidelines, and cite them as:

von Elm E, Altman DG, Egger M, Pocock SJ, Gotzsche PC, Vandenbroucke JP. The Strengthening the Reporting of Observational Studies in Epidemiology (STROBE) Statement: guidelines for reporting observational studies.

		Reporting Item	Page Number
**Title and abstract**			
Title	#1a	Indicate the study's design with a commonly used term in the title or the abstract	1
Abstract	#1b	Provide in the abstract an informative and balanced summary of what was done and what was found	3
**Introduction**			
Background / rationale	#2	Explain the scientific background and rationale for the investigation being reported	5
Objectives	#3	State specific objectives, including any prespecified hypotheses	6
**Methods**			
Study design	#4	Present key elements of study design early in the paper	6
Setting	#5	Describe the setting, locations, and relevant dates, including periods	7-8

For peer review only

		of recruitment, exposure, follow-up, and data collection	
Eligibility criteria	#6a	Give the eligibility criteria, and the sources and methods of selection of participants. Describe methods of follow-up.	9
Eligibility criteria	#6b	For matched studies, give matching criteria and number of exposed and unexposed	NA
Variables	#7	Clearly define all outcomes, exposures, predictors, potential confounders, and effect modifiers. Give diagnostic criteria, if applicable	9-10
Data sources / measurement	#8	For each variable of interest give sources of data and details of methods of assessment (measurement). Describe comparability of assessment methods if there is more than one group. Give information separately for for exposed and unexposed groups if applicable.	8-9
Bias	#9	Describe any efforts to address potential sources of bias	NA
Study size	#10	Explain how the study size was arrived at	9
Quantitative variables	#11	Explain how quantitative variables were handled in the analyses. If applicable, describe which groupings were chosen, and why	9
Statistical methods	#12a	Describe all statistical methods, including those used to control for confounding	
8-11			
Statistical methods	#12b	Describe any methods used to examine subgroups and interactions	NA
Statistical methods	#12c	Explain how missing data were addressed	NA
Statistical methods	#12d	If applicable, explain how loss to follow-up was addressed	NA
Statistical methods	#12e	Describe any sensitivity analyses	
11			
**Results**			
Participants	#13a	Report numbers of individuals at each stage of study—eg numbers potentially eligible, examined for eligibility, confirmed eligible,	11

For peer review only

		included in the study, completing follow-up, and analysed. Give information separately for for exposed and unexposed groups if applicable.	
Participants	#13b	Give reasons for non-participation at each stage	NA
Participants	#13c	Consider use of a flow diagram	
NA			
Descriptive data	#14a	Give characteristics of study participants (eg demographic, clinical, social) and information on exposures and potential confounders. Give information separately for exposed and unexposed groups if applicable.	NA
Descriptive data	#14b	Indicate number of participants with missing data for each variable of interest	
NA			
Descriptive data	#14c	Summarise follow-up time (eg, average and total amount)	
11			
Outcome data	#15	Report numbers of outcome events or summary measures over time. Give information separately for exposed and unexposed groups if applicable.	
12			
Main results	#16a	Give unadjusted estimates and, if applicable, confounder-adjusted estimates and their precision (eg, 95% confidence interval). Make clear which confounders were adjusted for and why they were included	12
Main results	#16b	Report category boundaries when continuous variables were categorized	NA
Main results	#16c	If relevant, consider translating estimates of relative risk into absolute risk for a meaningful time period	
NA			
Other analyses	#17	Report other analyses done—eg analyses of subgroups and interactions, and sensitivity analyses	11

Discussion

For peer review only

Key results	#18	Summarise key results with reference to study objectives	12
Limitations	#19	Discuss limitations of the study, taking into account sources of potential bias or imprecision. Discuss both direction and magnitude of any potential bias.	12-13
Interpretation	#20	Give a cautious overall interpretation considering objectives, limitations, multiplicity of analyses, results from similar studies, and other relevant evidence.	12-14
Generalisability	#21	Discuss the generalisability (external validity) of the study results	13-15
**Other Information**			
Funding	#22	Give the source of funding and the role of the funders for the present study and, if applicable, for the original study on which the present article is based	2

The STROBE checklist is distributed under the terms of the Creative Commons Attribution License CC-BY. This checklist was completed on 16. May 2023 using https://www.goodreports.org/, a tool made by the EQUATOR Network in collaboration with Penelope.ai

For peer review only

BMJ Open

Quantifying the mental health and economic impacts of prospective Universal Basic Income schemes among young people in the UK: a microsimulation modelling study

Journal:	*BMJ Open*
Manuscript ID	bmjopen-2023-075831.R1
Article Type:	Original research
Date Submitted by the Author:	06-Sep-2023
Complete List of Authors:	Chen, Tao; University of York<br>Reed, Howard; Landman Economics Ltd; Northumbria University, Department of Social Sciences<br>Mujica, Fiorella Parra; Erasmus University Rotterdam<br>Johnson, Elliott; Northumbria University, Social Work, Education and Community Wellbeing<br>Johnson, Matthew; Northumbria University, Department of Social Sciences<br>O'Flaherty, M; University of Liverpool, Department of Public Health, Policy & Systems<br>Collins, Brendan; University of Liverpool, Department of Public Health, Policy and Systems<br>Kypridemos, Christodoulos ; University of Liverpool, Department of Public Health, Policy & Systems
**Primary Subject Heading**:	Mental health
Secondary Subject Heading:	Health economics, Mental health, Health policy, Public health
Keywords:	MENTAL HEALTH, Depression & mood disorders < PSYCHIATRY, Anxiety disorders < PSYCHIATRY, PUBLIC HEALTH, HEALTH ECONOMICS

SCHOLARONE™
Manuscripts

I, the Submitting Author has the right to grant and does grant on behalf of all authors of the Work (as defined in the below author licence), an exclusive licence and/or a non-exclusive licence for contributions from authors who are: i) UK Crown employees; ii) where BMJ has agreed a CC-BY licence shall apply, and/or iii) in accordance with the terms applicable for US Federal Government officers or employees acting as part of their official duties; on a worldwide, perpetual, irrevocable, royalty-free basis to BMJ Publishing Group Ltd ("BMJ") its licensees and where the relevant Journal is co-owned by BMJ to the co-owners of the Journal, to publish the Work in this journal and any other BMJ products and to exploit all rights, as set out in our licence.

The Submitting Author accepts and understands that any supply made under these terms is made by BMJ to the Submitting Author unless you are acting as an employee on behalf of your employer or a postgraduate student of an affiliated institution which is paying any applicable article publishing charge ("APC") for Open Access articles. Where the Submitting Author wishes to make the Work available on an Open Access basis (and intends to pay the relevant APC), the terms of reuse of such Open Access shall be governed by a Creative Commons licence – details of these licences and which Creative Commons licence will apply to this Work are set out in our licence referred to above.

Other than as permitted in any relevant BMJ Author's Self Archiving Policies, I confirm this Work has not been accepted for publication elsewhere, is not being considered for publication elsewhere and does not duplicate material already published. I confirm all authors consent to publication of this Work and authorise the granting of this licence.

For peer review only

Quantifying the mental health and economic impacts of prospective Universal Basic Income schemes among young people in the UK: a microsimulation modelling study

Authors:

Tao Chen[1], tao.chen@york.ac.uk

Howard Reed[2,3], howard.r.reed@gmail.com

Fiorella Parra Mujica[4], parramujica@eshpm.eur.nl

Elliott Johnson[3], elliott.johnson@northumbria.ac.uk

Matthew Johnson[3], matthew7.johnson@northumbria.ac.uk

Martin O'Flaherty[5], moflaher@liverpool.ac.uk

Brendan Collins[5], brenc@liverpool.ac.uk

Chris Kypridemos[5], C.Kypridemos@liverpool.ac.uk

1. Centre for Health Economics, University of York
2. Landman Economics Ltd, http://www.landman-economics.co.uk/
3. Department of Social Work, Education and Community Wellbeing, Northumbria University, UK
4. Erasmus School of Health Policy and Management (ESHPM), Erasmus University Rotterdam, Netherlands
5. Department of Public Health, Policy & Systems, University of Liverpool, UK

For peer review only

Correspondence to:

Chris Kypridemos, PhD

Department of Public Health, Policy & Systems, Institute of Population Health, Whelan Building, Quadrangle, Liverpool L69 3GB, UK

Abstract:

Objective

Universal Basic Income (UBI) – a largely unconditional, regular payment to all adults to support basic needs – has been proposed as a policy to increase the size and security of household incomes and promote mental health. We aimed to quantify its long-term impact on mental health among young people in England.

Methods

We produced a discrete-time dynamic stochastic microsimulation that models a close-to-reality open cohort of synthetic individuals (2010-2030) based on data from Office for National Statistics and Understanding Society. Three UBI scheme scenarios were simulated: Scheme 1 – Starter (per week): £41 per child; £63 per adult over 18 and under 65; £190 per adult aged 65+; Scheme 2 – Intermediate (per week): £63 per child; £145 per adult under 65; £190 per adult aged 65+; Scheme 3 – Minimum Income Standard level (per week): £95 per child; £230 per adult under 65; £230 per adult aged 65+. We reported cases of anxiety and depression prevented or postponed and cost savings. Estimates are rounded to the 2nd significant digit.

Results

Scheme 1 could prevent or postpone 200,000(95% uncertainty interval: 180,000 - 210,000) cases of anxiety and depression from 2010-2030. This would increase to 420,000(400,000 - 440,000) for Scheme 2 and 550,000(520,000 - 570,000) for Scheme 3. Assuming that 50% of cases are diagnosed and treated, Scheme 1 could save £330m(£280m - £390m) to NHS and personal social services (PSS), over the same period, with Scheme 2(£710 million[£640m -

For peer review only

£790m]) or Scheme 3(£930 million[£850m - £1000m]) producing more considerable savings. Overall, total cost savings (including NHS, PSS and patients' related costs) would range from £1.5 billion (£1.2b - £1.8b) for Scheme 1 to £4.2 billion (£3.7b - £4.6b) for Scheme 3.

Conclusion

Our modelling suggests that UBI could substantially benefit young people's mental health, producing substantial health-related cost savings.

Keywords: Universal Basic Income; Mental Health; Prevention; Microsimulation Modelling

Strengths and limitations of this study

- A microsimulation based on real-world data from the Understanding Society longitudinal study, assuming a causal relationship between income and anxiety and depression and total risk reversibility.
- Mental health and economic potential impacts of universal basic income implementations are explored.
- Limited to pre-pandemic data, so impacts of cost of living crisis and other changes since the pandemic may change the results.
- It does not include the impact of people moving into higher tax brackets ('fiscal drag').
- The modelled effect size of the universal basic income is based on observational data (Understanding Society) that may suffer from selection bias, misclassification, survivorship bias, and reverse causality.

Introduction

There is a crisis in mental health among young people, which will have long-term impacts on well-being and development. Between 1995 and 2014, the proportion of 16- to 24-year-olds in the United Kingdom (UK) reporting a longstanding mental health condition increased from 0.6% to 5.9% [1]. A meta-analysis including 11 high-income countries indicated that one in eight children have mental disorders [2]. Unfortunately, this problem may have been exacerbated since the recent austerity period in the UK [3] and further magnified during the COVID pandemic [4]. Currently, it is estimated that childhood mental disorders are the leading cause of childhood disability globally[5] and incur considerable social and economic burdens to the healthcare system and families [6, 7].

Previous studies have found that adverse economic conditions could negatively affect mental health in children and young people [8-10]. Our previous analysis of Understanding Society data for young people aged 16-24 from UK households went further, showing a dose-response effect[11]. Young people living in households within the lowest net equivalised income quintile group had a higher probability than the second lowest quintile group of reporting clinically significant symptoms of anxiety and depression; the second lowest had a higher probability than the middle quintile group and so on up the income scale.

To address this public health concern, many approaches have been proposed to promote mental health and prevent mental disorders[12, 13]. However, these reactive policies have often focused on individual-level interventions such as improving coping strategies and increasing the efficiency of services. At the same time, interest is growing in addressing the social causes of anxiety and depression. A large body of evidence indicates that social determinants strongly affect those conditions: income, wealth, education, social capital and opportunity [10, 14-16]. One proposed means of addressing these issues, which is increasingly gaining support from various organisations, policymakers and politicians, is Universal Basic Income (UBI), a largely

For peer review only

unconditional, regular payment to all permanent residents to support basic needs. Johnson and colleagues have set out a theoretical model of its impact that indicates that UBI can mitigate social determinants of health by reducing poverty, mitigating inequality and fostering long-term, health-promoting behaviour[14, 17].

Previous modelling has examined the potential costs and benefits of mental health interventions to prevent or treat anxiety, depression, bipolar disorder, and suicide among adolescents[13] or by comparing cognitive behavioural therapy and selective serotonin reuptake inhibitors for major depression in children and adolescents[18]. However, no study assessed the long-term impact of UBI on mental health in children and young people.

This study aimed to quantify the potential impacts of three prospective UBI schemes on the mental health of young people and the associated economic burden during the 2010-2030 period in the UK.

Methods

There are multiple pathways for a UBI scheme to impact health. In Figure 1, we present a comprehensive model of impact with three distinct but perhaps synergistic biopsychosocial pathways to impact health, including mental health (for more details, please refer to Johnson and colleagues[17]). The present study examines the impact of changes in income, specifically on anxiety and depression. This focuses largely on the pathway associated with poverty reduction. However, the redistributive effects of the schemes modelled may also track the impacts of reduction in inequality. Larger incomes are also often more predictable. The data that informed our models are observational; therefore, it is difficult to disentangle and quantify the pathways in our analysis. Experimental data and qualitative analysis would be required to establish the relative impacts of each pathway.

For peer review only

Our study used two microsimulations in a hybrid serial modelling arrangement to simulate three UBI scheme scenarios and estimate the prevalence of anxiety and depression and consequent deaths under the counterfactual net equivalised household income distributions.

The three UBI scheme scenarios were broadly designed to provide pathways towards attaining the Minimum Income Standard (MIS) with income distributions microsimulated using the Landman Economics Tax-Transfer Model (first microsimulation in the serial arrangement). MIS is the income needed by different types of households to reach a socially acceptable living standard, as determined by members of the public with support from experts[19]. The three schemes are detailed below, and Table 1 outlines the cost of each UBI scheme used in the paper showing that they are all fiscally neutral (at least in terms of first-round static effects):

Scheme 1 – Starter (per week): £41 per child; £63 per adult over 18 and under 65; £190 per adult aged 66+

Scheme 1 is a realistic 'starter' scheme with relatively low payments for working age adults and children, but payments for pensioners which are above the level of the current UK state retirement pension for a pensioner with a full record of National Insurance contributions during working life.

Scheme 2 – Intermediate (per week): £63 per child; £145 per adult under 65; £190 per adult aged 66+

Scheme 2 is a mid-point between the Schemes 1 and 3.

Scheme 3 – MIS level (per week): £95 per child; £230 per adult under 65; £230 per adult aged 66+

Scheme 3 ensures that all families reach the MIS level.

Each of the above schemes is intended to meet the following conditions:

For peer review only

1. UBI would be paid to eligible residents without condition, raising the incomes of the lower income groups.

2. UBI would reduce the percentage gap between the top and bottom income groups through fiscal reform, be high enough to make a material difference in people's lives and raise the level of universality in the social security system, thus reducing reliance on means-testing.

3. UBI would be affordable (although this depends on how this is defined);

4. UBI would minimise losses for low-income households, minimise the amount of disruption involved in moving to a new income support system, and enjoy broad public support. For instance, these schemes have been found to enjoy support among critical "red wall" voters[20].

Table 1. Fiscal costings of the three UBI scheme scenarios (£bn)

**All costs/savings in £bn**	Scheme 1	Scheme 2	Scheme 3
Gross cost of UBI	274.4	464.3	677.5
**Benefit savings:**			
Abolition of child benefit	10.6	10.6	10.6
Abolition of state pension	96.9	96.9	96.9
Reduction in Universal Credit/ legacy benefits	7.1	37.4	59.6
**Total savings**	**114.6**	**144.9**	**167.1**
**Tax changes:**			
Reduction of personal allowance to £750	90.9	90.9	90.9
National Insurance changes	54.4	-78.2	-78.2
Income tax rate increases	14.7	306.9	497.7
**Total tax increases**	**160.0**	**319.7**	**510.5**
**Net cost**	**-0.2**	**-0.3**	**-0.1**

For peer review only

Household income modelling

The Landman Economics Tax Transfer Model was used with Waves 1 to 10 (inclusive) of Understanding Society data to microsimulate the UBI payments in Schemes 1, 2 and 3 and corresponding packages of increases to tax (income tax and National Insurance contributions) required to achieve fiscal balance for each scheme, taking into account reductions in payments of existing benefits[21]. The main non-means-tested benefits in the UK benefits system (Child Benefit and the State Pension) are replaced by the UBI in each scheme. UBI payments are counted as unearned income to calculate Universal Credit (a means-tested transfer payment for people on low incomes in the UK benefits system). So UBI payments replace Universal Credit payments one-for-one for low-income individuals (although a small disregard is applied for schemes 1 and 2 so that low-income individuals and families are better off under UBI than the baseline system. In Scheme 3, UBI payments are sufficiently high that no disregard is necessary). Note that fiscal balancing for each scheme (i.e. ensuring that the increase in tax revenue approximately matches the cost of UBI expenditure, net of any reductions in other benefits) is done statically in the model, not taking account of any behavioural changes in response to the receipt of UBI payments.

The level of payments in each of the schemes is based on existing analysis by Reed and colleagues [21] using data from the UK Family Resources Survey (FRS) for the 2019/20 fiscal year. This overlaps with the interview dates for Understanding Society Wave 10, so the same level of payments is used for Wave 10 as for the FRS analysis. For earlier waves of Understanding Society, the UBI payments are deflated using the UK Consumer Prices Index. The income tax and National Insurance increases are adjusted in each wave to ensure approximate fiscal balance and to compensate for any change in real incomes between waves of Understanding Society. Therefore, to ensure that the combination of the introduction of UBI

For peer review only

and changes to the benefits system, and the accompanying income tax and NICs increases, are approximately fiscally neutral in each wave.

Health and disease costs impact modelling

The second microsimulation in the serial arrangement was a discrete-time dynamic stochastic microsimulation that used the output of the first microsimulation (changes in the distribution of the equivalised household income) and translated into changes in the prevalence of anxiety and depression and the consecutive disease costs. Specifically, the second microsimulation models a close-to-reality open cohort of synthetic individuals (starting at 90,000) representing individuals aged 14-24 in the UK between 2010 and 2030. Their rates of fertility, mortality, and migration were driven by Office for National Statistics (ONS) estimates and projections[22]. At the same time, ethnicity, whether born in the UK, highest educational attainment and marital status were informed by waves 1-10 of the Understanding Society: the UK Household Longitudinal Study[23]. We simulated the prevalence of anxiety and depression using the SF-12 Mental Component Summary measure with a clinical threshold score of ≤45.6. Our simulation was based on all attributes above, including equivalised household income, based on evidence from Parra-Mujica and colleagues[11], assuming a causal relationship between income and anxiety and depression and total risk reversibility. For all the attributes described above, we fitted logistic regression models to the Understanding Society data and predicted from them to allocate the attributes of the synthetic individuals. We further assumed that the observed increasing trend of anxiety and depression prevalence would plateau after 2019 to avoid an unrealistic increase over time. We did the same for the equivalised household income trends post 2019.

So, when a synthetic individual enters the simulation, their age and sex are defined based on ONS estimates. Then their ethnicity (white/other), place of birth (UK/elsewhere), education,

For peer review only

and marital status are estimated using regression models fitted in Understanding Society. Age, sex, and year are predictors in all these regressions. Additionally, place of birth was a predictor for ethnicity; ethnicity and place of birth were predictors for education, and all previous attributes were predictors for marital status. Finally, using the Landman Economics Tax Transfer Model, the household equivalised income is estimated based on all previously simulated attributes and the scenario (baseline or one of the UBI Schemes). For every simulated year, the age of synthetic individuals increases by one and education, marital status and household equivalised income attributes are updated. The prevalence of anxiety and depression is estimated for each synthetic individual based on all previous attributes using a logistic regression fitted in Understanding Society data.

In our microsimulation, we also modelled reductions in cause-excess deaths based on relative risks (RR) identified in observational data from Denmark by Meier and colleagues[24]. To account for the fact that our case definition of anxiety and depression, based on a self-reported measure (SF-12 MCS), might also include less severe cases compared to the clinically diagnosed cases in the study by Meier and colleagues and reflect the uncertainty of this parameter, we formed a beta-PERT distribution[25] based on estimates from the study mentioned above. We used the low 95% confidence interval of the fully adjusted all-cause mortality RR (1.56) reported for individuals diagnosed with anxiety disorders as the minimum for our beta-PERT distribution, the anxiety disorders RR for ages <30 (4.4) as the mode of the distribution, and the dual diagnosis of anxiety and depression RR for ages < 30 (6.9) as the maximum of the distribution. These estimates were comparable to published estimates from Sweden, albeit using slightly different case definitions [26].

Costs

For peer review only

Costs for anxiety and depression treatment were informed by the usual care arm of the CADET randomised control trial[27]. We estimated and report two different cost perspectives 1) the UK NHS and Personal Social Services (Third Party Payer) perspective, and 2) A broader perspective that included resource use from primary/Community Care (e.g., GP, Mental Health worker, Social worker), Secondary Care (e.g., Hospital admissions, Psychiatric rehab ward, Outpatient appointment, social care (e.g., Daycare centre, drop in a club), informal care from friends/relatives (e.g., Hours per week help from friends/relatives), patient other costs (e.g., OTC medications, Travel costs) to estimate the total cost of anxiety and depression. We inflated all costs to mid-2015 British pounds using the Consumer Price Index and did not apply an annual discount rate for costs occurring in the past or future. Finally, we used the reported mean and standard deviation of the costs to form Gamma distributions using the method of moments and capture the uncertainty of the inputs. We further assumed that only half of the synthetic individuals that reported symptoms of anxiety or depression would seek treatment and thus incur healthcare costs. This assumption was roughly informed by the Adult Psychiatric Morbidity Survey [28].

Uncertainty and sensitivity analysis

In all reported figures, we ensured that we captured the uncertainty of the outputs. The microsimulation used a second-order Monte Carlo with 200 iterations for the outer loop and ~90,000 iterations for the inner loop to propagate the uncertainty of the inputs to the outputs[29]. We summarised the uncertainty of the outputs by reporting the median and 95% Uncertainty Interval (UI) of their respective distributions. The three main sources of uncertainty in the model are 1) the strength of the relation between equivalised household income and anxiety and depression, 2) the excess mortality risk from anxiety and depression, 3) the disease costs, and 4) the individual heterogeneity from the modelled attributes of the synthetic individuals.

For peer review only

The outputs from the model are case-years of anxiety and depression prevented or postponed (CYPP), deaths prevented or postponed (DPP), disease costs from the NHS and personal social services perspective, and total disease costs. All our estimates are rounded to the 2nd significant digit.

Patient and public involvement

None.

Results

The UK population between 14 and 24 years old was projected to increase from approximately 9 million in 2010 to 9.6 million in 2030. Of those, about 2 million would experience symptoms of anxiety and depression in 2010; this was projected to more than double to 4.1 million by 2030. Anxiety and depression were more prevalent in females, about 46%, versus about 30% in males, and their prevalence increased with age. It was also slightly more prevalent among non-White and those born in the UK.

The model estimated that approximately 200,000 (95% UI: 180,000 - 210,000) cases of anxiety and depression could be prevented or postponed in Scheme 1 from 2010-2030. The effectiveness would increase to 420,000 (95% UI: 400,000 - 440,000) for Scheme 2 and 550,000 (95% UI: 520,000 - 570,000) for Scheme 3. In relative terms, these represent approximately 0.028% (95% UI: 0.026% - 0.030%) of all case years with anxiety and depression for Scheme 1, 0.059% (95% UI: 0.056% - 0.063%) for Scheme 2 and 0.077% (95% UI: 0.074% - 0.081%) for Scheme 3. Correspondingly, 110 (95% UI: 0 - 430), 320 (95% UI: 0 - 640) and 420 (95% UI: 100 - 770) deaths would be prevented or postponed for the three Schemes, respectively.

Table 2 shows the NHS and personal social services cost savings and total cost savings. Overall, the total cost saving, including NHS, personal social services and patient-related costs,

For peer review only

would range from £1.5 billion (£1.2b - £1.8b) for Scheme 1 to £4.2 billion (£3.7b - £4.6b) for Scheme 3.

Table 2. Modelling results estimating disease cost savings from different perspectives

Schemes	NHS and personal social services cost savings over 2010-2030, assuming 50% of cases diagnosed and treated	Total cost savings over 2010-2030, assuming 50% of cases diagnosed and treated
**Scheme 1**	£330 million (£280m - £390m)	£1.5 billion (£1.2b - £1.8b)
**Scheme 2**	£710 million (£640m - £790m)	£3.2 billion (£2.8b - £3.6b)
**Scheme 3**	£930 million (£850m - £1000m)	£4.2 billion (£3.7b - £4.6b)

Discussion

Our findings indicate the mental health impact that UBI could have on a specific age group through a pathway of increased income. Despite the limited scope of the present modelling study, it is clear that the potential is substantial and significant. Over 21 years, 200 to 550 thousand cases of anxiety and depression could be prevented or postponed, saving £330m to £930m in health and social services costs. In reality, these are opportunity costs rather than cashable savings; most NHS costs are fixed staffing costs. Since demand typically outstrips supply for NHS mental health services –the prevented cases of anxiety and depression will mean that other people will benefit by receiving treatment more quickly [30].

To our knowledge, this is the first study to model the health and disease cost impact of UBI among young people. Previous modelling mainly focused on assessing mental health prevention through trial-based economic evaluation[18, 27] but was subject to inadequate patient follow-up and not capturing the final health outcomes. However, our model-based

For peer review only

design is fundamental in an economic evaluation of mental health prevention due to its advantages, including the ability to consider all relevant prospective policy alternatives, including evidence not often collected in trials, and extrapolate beyond the usually short-term horizon of empirical studies [31].

Our modelling exercise assumes that low income is causally related to anxiety and depression and that increasing income can fully reverse the risk. The association between income and mental health has been shown in experimental and observational studies [10, 15]. However, the heterogeneity of cash transfer schemes and other policies intended to redistribute income and reported mental health outcomes makes evidence synthesis difficult. Large, representative trials of UBI that capture comprehensive and comparable data in the real world are crucial[14].

In the future, it is imperative to develop models that comprehensively capture the health impact of income changes across the entire population and all major disease types. These models should incorporate quality of life measures such as quality-adjusted life years (QALYs) gained, and their value could be evaluated based on NICE or UK Treasury valuations. Such an approach would enable assessment of the potential cost savings that could be achieved through improved health outcomes under a UBI policy. Moreover, the additional equity and well-being benefits of UBI, which are not fully captured through a 'burden of disease' perspective, may further offset some of the financial burdens associated with implementing such policies.

Regarding policy implications, the present study provides evidence that UBI can produce health benefits for young people over a medium-term time horizon. This is useful evidence for the basic income trial currently underway in Wales, in which care leavers are offered a basic income of £1,600 per month (higher than Scheme 3 in the present study)[32]. Care leavers have rates of mental health problems that are up to six times that of the non-care exposed population[33], so there is potential for a basic income for care leavers to have a greater relative

effect in this group, depending on how much mental health problems are related to income in this population. There is also a basic income pilot in Santa Clara County (California, USA), where people leaving foster care at age 25 receive $1000 a month. Including common outcome measures such as SF-12 in these real-world pilots would provide further data to compare with the results of this study and enable further microsimulation modelling.

The concept of a guaranteed minimum income the state provides to all permanent residents is gaining traction across the political spectrum. Even the conservative-leaning UK think tank Bright Blue recently called for “the establishment of a new ‘minimum living’ income” [34], although largely within the UK welfare system as it is currently constituted. A scoping review of the public health effects of interventions resembling basic income found “modest to strong positive effects on several health outcomes, including low birth weight, infant obesity, adult and child mental health, service use, and nutrition” [35]. An evidence synthesis based on several studies of basic income programmes found that, overall, basic income improved mental health, with mediating factors being increased free time, hope for the future, and reduced stigma[36].

Our modelling exercise has some limitations. First, when setting the UBI payment levels and the income tax thresholds in the reform schemes, we assumed that both are CPI-uprated between Understanding Society Waves 1 and 10. This means that UBI payments for each adult and child remain constant in real terms from year to year. We made the same assumption about tax. However, this fails to account for the fact that real earnings grew in most years between 2010 and 2019, resulting in a process known as “fiscal drag” (taxpayers tending to move into higher marginal rate brackets) that would gradually decrease the impact of the UBI schemes. To minimise this bias and considering the turbulent period since 2020, we did not model trends in equivalised household income post 2019. Second, all the data we used were from the years before the COVID-19 pandemic and the post-pandemic cost-of-living crisis. Therefore, the

For peer review only

trends we modelled may not be indicative of the post-pandemic period up to 2030. Specifically, the pandemic may increase the prevalence of anxiety and depression in the population and further limit access to appropriate treatments and support. Furthermore, the post-pandemic cost-of-living crisis and the high-inflation period may compress family incomes and accelerate the mental health crisis. These limitations make our modelled estimates conservative and research on UBI policies more relevant than ever.

However, there are also potential sources of bias whose direction and magnitude are unclear. For instance, the modelled effect size of UBI schemes is based on observational data (Understanding Society) that may suffer from selection bias, misclassification, survivorship bias, and reverse causality. Although, a recent meta-analysis found that the effect of income changes on mental health was reported as larger when experimental studies were exclusively considered versus when only observational studies were considered.[10] Finally, our modelling does not include wider potentially unintended consequences that the restructuring of the income redistribution system might cause to the economy.

Conclusions

In summary, the present study suggests that UBI could substantially improve mental health in young people, reduce costs related to the NHS, personal social services and patients, and reduce premature mortality. These findings add to the growing body of evidence supporting the potential for UBI to improve population health.

Funding

This research was funded by a Wellcome Trust Discretionary Award (223553/Z/21/Z):

‘Assessing the prospective impacts of Universal Basic Income on anxiety and depression

For peer review only

among 14–24-year-olds’. The funder had no role in study design, data collection and analysis, decision to publish, or preparation of the manuscript.

Competing interests

BC works part-time for Welsh Government; this does not represent any views of the Welsh Government. All other co-authors declare no conflict of interest.

Contributors

MJ and EJ conceptualised the study; TC and FPM analysed the data; HR developed and performed the UBI modelling; CK developed and performed the health modelling and supervised all the analysis; TC produced the first draft; all authors (MJ, EJ, TC, FPM, HR, CK, MOF, BC) interpreted the results and critically revised the manuscript.

Data availability statement

This is a modelling study that generated no new empirical data. Understanding Society is available through the UK data service (https://ukdataservice.ac.uk/).

Ethics approval

This study is a modelling study that did not include human participants, therefore ethics approval was not required.

For peer review only

References

1. Pitchforth J, Fahy K, Ford T, et al. Mental health and well-being trends among children and young people in the UK, 1995-2014: analysis of repeated cross-sectional national health surveys. *Psychol Med* 2019;49(8):1275-85. doi: 10.1017/S0033291718001757 [published Online First: 2018/09/12]
2. Barican JL, Yung D, Schwartz C, et al. Prevalence of childhood mental disorders in high-income countries: a systematic review and meta-analysis to inform policymaking. *Evid Based Ment Health* 2022;25(1):36-44. doi: 10.1136/ebmental-2021-300277 [published Online First: 20210719]
3. Thomson RM, Katikireddi SV. Mental health and the jilted generation: Using age-period-cohort analysis to assess differential trends in young people's mental health following the Great Recession and austerity in England. *Soc Sci Med* 2018;214:133-43. doi: 10.1016/j.socscimed.2018.08.034 [published Online First: 20180829]
4. Ma L, Mazidi M, Li K, et al. Prevalence of mental health problems among children and adolescents during the COVID-19 pandemic: A systematic review and meta-analysis. *J Affect Disord* 2021;293:78-89. doi: 10.1016/j.jad.2021.06.021 [published Online First: 2021/06/27]
5. Erskine HE, Moffitt TE, Copeland WE, et al. A heavy burden on young minds: the global burden of mental and substance use disorders in children and youth. *Psychol Med* 2015;45(7):1551-63. doi: 10.1017/S0033291714002888 [published Online First: 2014/12/24]
6. Lim KL, Jacobs P, Ohinmaa A, et al. A new population-based measure of the economic burden of mental illness in Canada. *Chronic Dis Can* 2008;28(3):92-8. [published Online First: 2008/03/18]
7. Fineberg NA, Haddad PM, Carpenter L, et al. The size, burden and cost of disorders of the brain in the UK. *J Psychopharmacol* 2013;27(9):761-70. doi: 10.1177/0269881113495118 [published Online First: 2013/07/26]
8. Golberstein E, Gonzales G, Meara E. How do economic downturns affect the mental health of children? Evidence from the National Health Interview Survey. *Health Econ* 2019;28(8):955-70. doi: 10.1002/hec.3885 [published Online First: 2019/06/06]
9. Pfoertner TK, Rathmann K, Elgar FJ, et al. Adolescents' psychological health complaints and the economic recession in late 2007: a multilevel study in 31 countries. *Eur J Public Health* 2014;24(6):961-7. doi: 10.1093/eurpub/cku056 [published Online First: 2014/05/27]
10. Thomson RM, Igelström E, Purba AK, et al. How do income changes impact on mental health and wellbeing for working-age adults? A systematic review and meta-analysis. *The Lancet Public Health* 2022;7(6):e515-e28. doi: 10.1016/s2468-2667(22)00058-5
11. Parra-Mujica F, Johnson, E.A., Cookson, R., Reed, H. & Johnson, M.T. Understanding the relationship between socioeconomic status, income and mental health among 16- to 24-year-olds: Analysis of 10 waves (2009-2020) of Understanding Society to enable modelling of income interventions. *PLOS ONE* 2022;18(2):e0279845. doi: 10.1371/journal.pone.0279845
12. Dunning DL, Griffiths K, Kuyken W, et al. Research Review: The effects of mindfulness-based interventions on cognition and mental health in children and adolescents - a meta-analysis of randomized controlled trials. *J Child Psychol Psychiatry* 2019;60(3):244-58. doi: 10.1111/jcpp.12980 [published Online First: 2018/10/23]
13. Skeen S, Laurenzi CA, Gordon SL, et al. Adolescent Mental Health Program Components and Behavior Risk Reduction: A Meta-analysis. *Pediatrics* 2019;144(2) doi: 10.1542/peds.2018-3488 [published Online First: 2019/07/03]
14. Johnson EA, Johnson, M.T., Kypridemos, C., Villadsen, A. & Pickett, K.E. Designing a generic, adaptive protocol resource for the measurement of health impact in cash transfer trials'. *Research Square* 2022
15. Romero J, Esopo, K., McGuire, J. & Haushofer. The Effect of Economic Transfers on Psychological Well-Being and Mental Health. *working paper* 2021

16. Kromydas T, Thomson RM, Pulford A, et al. Which is most important for mental health: Money, poverty, or paid work? A fixed-effects analysis of the UK Household Longitudinal Study. *SSM-population Health* 2021;15:100909.
17. Johnson MT, Johnson EA, Nettle D, et al. Designing trials of Universal Basic Income for health impact: identifying interdisciplinary questions to address. *Journal of Public Health* 2021;44(2):408-16. doi: 10.1093/pubmed/fdaa255
18. Haby MM, Tonge B, Littlefield L, et al. Cost-effectiveness of cognitive behavioural therapy and selective serotonin reuptake inhibitors for major depression in children and adolescents. *Aust N Z J Psychiatry* 2004;38(8):579-91. doi: 10.1080/j.1440-1614.2004.01421.x [published Online First: 2004/08/10]
19. Davis A, Hirsch, D., Padley, M. & Shepherd, C. A Minimum Income Standard for the United Kingdom in 2021: York: Joseph Rowntree Foundation 2021.
20. Johnson M, Nettle D, Johnson E, et al. Winning the vote with a universal basic income: Evidence from the 'red wall', 2022.
21. Reed HR, Johnson MT, Lansley S, et al. Universal Basic Income is affordable and feasible: evidence from UK economic microsimulation modelling. *Journal of Poverty and Social Justice* 2023;31(1):146-62. doi: 10.1332/175982721X16702368352393
22. 2020-based interim national population projections - Office for National Statistics [
23. Research IfSaE. Understanding Society, 2022.
24. Meier SM, Mattheisen M, Mors O, et al. Increased mortality among people with anxiety disorders: total population study. *Br J Psychiatry* 2016;209(3):216-21. doi: 10.1192/bjp.bp.115.171975 [published Online First: 2016/07/09]
25. Clark CE. Letter to the editor-the PERT model for the distribution of an activity time. *Operations Research* 1962;10(3):405-06. doi: 10/bg3bp2
26. Leone M, Kuja-Halkola R, Leval A, et al. Association of Youth Depression With Subsequent Somatic Diseases and Premature Death. *JAMA Psychiatry* 2021;78(3):302-10. doi: 10.1001/jamapsychiatry.2020.3786 [published Online First: 2020/12/10]
27. Green C, Richards DA, Hill JJ, et al. Cost-effectiveness of collaborative care for depression in UK primary care: economic evaluation of a randomised controlled trial (CADET). *PLoS One* 2014;9(8):e104225. doi: 10.1371/journal.pone.0104225 [published Online First: 2014/08/15]
28. Digital N. Adult Psychiatric Morbidity Survey: Survey of Mental Health and Wellbeing, England, 2014. 2014 [
29. Koerkamp BG, Stijnen T, Weinstein MC, et al. The combined analysis of uncertainty and patient heterogeneity in medical decision models. *Medical Decision Making* 2011;31(4):650-61. doi: 10/cdf2wf
30. Iqbal Z, Airey ND, Brown SR, et al. Waiting list eradication in secondary care psychology: Addressing a National Health Service blind spot. *Clinical Psychology & Psychotherapy* 2021;28(4):969-77. doi: https://doi.org/10.1002/cpp.2551
31. Ha NT, Huong NT, Anh VN, et al. Modelling in economic evaluation of mental health prevention: current status and quality of studies. *BMC Health Serv Res* 2022;22(1):906. doi: 10.1186/s12913-022-08206-9 [published Online First: 2022/07/14]
32. Government W. Basic income pilot for care leavers: overview of the scheme 2023 [updated 2023-02-15.
33. Butterworth S, Singh SP, Birchwood M, et al. Transitioning care-leavers with mental health needs: 'they set you up to fail!'. *Child Adolesc Ment Health* 2017;22(3):138-47. doi: 10.1111/camh.12171 [published Online First: 20160629]
34. Sarygulov A, Arslanagić-Wakefield P. Building up: The future of social security. London, UK: bright blue, 2023.
35. Gibson M, Hearty W, Craig P. The public health effects of interventions similar to basic income: a scoping review. *The Lancet Public Health* 2020;5(3):e165-e76. doi: 10.1016/S2468-2667(20)30005-0

For peer review only

36. Wilson N, McDaid S. The mental health effects of a Universal Basic Income: A synthesis of the evidence from previous pilots. *Soc Sci Med* 2021;287:114374. doi: 10.1016/j.socscimed.2021.114374 [published Online First: 20210907]

Figure legend

Figure 1. Universal Basic Income pathways to health and potential cost neutrality
Adapted from Johnson and colleagues[17].

For peer review only

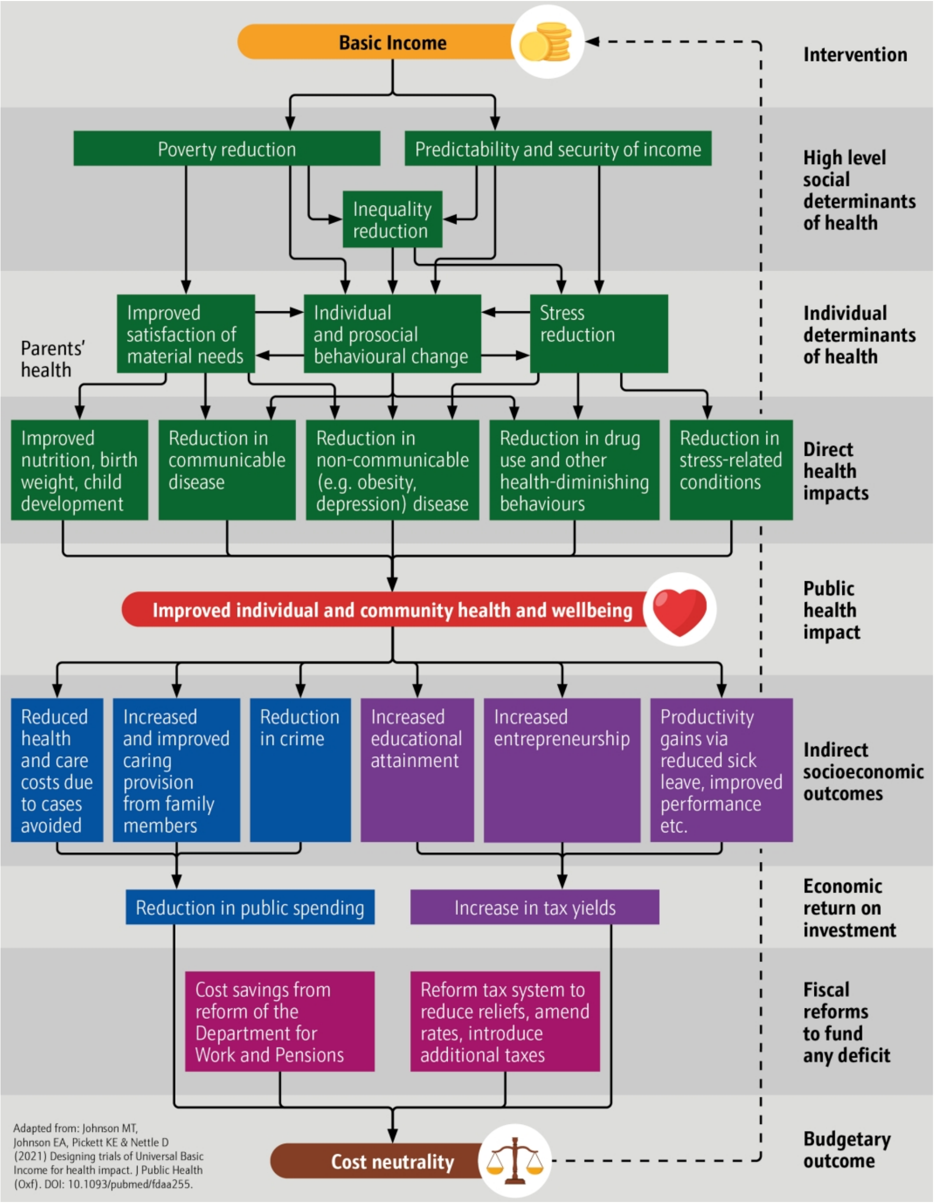

Figure 1: Universal Basic Income pathways to health and potential cost neutrality. Adapted from Johnson and colleagues.

481x618mm (118 x 118 DPI)

Reporting checklist for cohort study.

Based on the STROBE cohort guidelines.

Instructions to authors

Complete this checklist by entering the page numbers from your manuscript where readers will find each of the items listed below.

Your article may not currently address all the items on the checklist. Please modify your text to include the missing information. If you are certain that an item does not apply, please write "n/a" and provide a short explanation.

Upload your completed checklist as an extra file when you submit to a journal.

In your methods section, say that you used the STROBE cohortreporting guidelines, and cite them as:

von Elm E, Altman DG, Egger M, Pocock SJ, Gotzsche PC, Vandenbroucke JP. The Strengthening the Reporting of Observational Studies in Epidemiology (STROBE) Statement: guidelines for reporting observational studies.

For peer review only

		Reporting Item	Page Number
**Title and abstract**			
Title	#1a	Indicate the study's design with a commonly used term in the title or the abstract	1
Abstract	#1b	Provide in the abstract an informative and balanced summary of what was done and what was found	3
**Introduction**			
Background / rationale	#2	Explain the scientific background and rationale for the investigation being reported	5
Objectives	#3	State specific objectives, including any prespecified hypotheses	6
**Methods**			
Study design	#4	Present key elements of study design early in the paper	6
Setting	#5	Describe the setting, locations, and relevant dates, including periods	7-8

		of recruitment, exposure, follow-up, and data collection	
Eligibility criteria	#6a	Give the eligibility criteria, and the sources and methods of selection of participants. Describe methods of follow-up.	9
Eligibility criteria	#6b	For matched studies, give matching criteria and number of exposed and unexposed	NA
Variables	#7	Clearly define all outcomes, exposures, predictors, potential confounders, and effect modifiers. Give diagnostic criteria, if applicable	9-10
Data sources / measurement	#8	For each variable of interest give sources of data and details of methods of assessment (measurement). Describe comparability of assessment methods if there is more than one group. Give information separately for for exposed and unexposed groups if applicable.	8-9
Bias	#9	Describe any efforts to address potential sources of bias	NA
Study size	#10	Explain how the study size was arrived at	9
Quantitative variables	#11	Explain how quantitative variables were handled in the analyses. If applicable, describe which groupings were chosen, and why	9
Statistical methods	#12a	Describe all statistical methods, including those used to control for confounding	
8-11			
Statistical methods	#12b	Describe any methods used to examine subgroups and interactions	NA
Statistical methods	#12c	Explain how missing data were addressed	NA
Statistical methods	#12d	If applicable, explain how loss to follow-up was addressed	NA
Statistical methods	#12e	Describe any sensitivity analyses	
11			
**Results**			
Participants	#13a	Report numbers of individuals at each stage of study—eg numbers potentially eligible, examined for eligibility, confirmed eligible,	11

For peer review only

		included in the study, completing follow-up, and analysed. Give information separately for for exposed and unexposed groups if applicable.	
Participants	#13b	Give reasons for non-participation at each stage	NA
Participants	#13c	Consider use of a flow diagram	
NA			
Descriptive data	#14a	Give characteristics of study participants (eg demographic, clinical, social) and information on exposures and potential confounders. Give information separately for exposed and unexposed groups if applicable.	NA
Descriptive data	#14b	Indicate number of participants with missing data for each variable of interest	
NA			
Descriptive data	#14c	Summarise follow-up time (eg, average and total amount)	
11			
Outcome data	#15	Report numbers of outcome events or summary measures over time. Give information separately for exposed and unexposed groups if applicable.	
12			
Main results	#16a	Give unadjusted estimates and, if applicable, confounder-adjusted estimates and their precision (eg, 95% confidence interval). Make clear which confounders were adjusted for and why they were included	12
Main results	#16b	Report category boundaries when continuous variables were categorized	NA
Main results	#16c	If relevant, consider translating estimates of relative risk into absolute risk for a meaningful time period	
NA			
Other analyses	#17	Report other analyses done—eg analyses of subgroups and interactions, and sensitivity analyses	11

Discussion

For peer review only

Key results	#18	Summarise key results with reference to study objectives	12
Limitations	#19	Discuss limitations of the study, taking into account sources of potential bias or imprecision. Discuss both direction and magnitude of any potential bias.	12-13
Interpretation	#20	Give a cautious overall interpretation considering objectives, limitations, multiplicity of analyses, results from similar studies, and other relevant evidence.	12-14
Generalisability	#21	Discuss the generalisability (external validity) of the study results	13-15
**Other Information**			
Funding	#22	Give the source of funding and the role of the funders for the present study and, if applicable, for the original study on which the present article is based	2

The STROBE checklist is distributed under the terms of the Creative Commons Attribution License CC-BY. This checklist was completed on 16. May 2023 using https://www.goodreports.org/, a tool made by the EQUATOR Network in collaboration with Penelope.ai

For peer review only
